# Morphometric Analysis of the Maxillary Sinus and Its Implications for Sinus Augmentation Surgery in an Eastern Indian Population

**DOI:** 10.7759/cureus.87809

**Published:** 2025-07-13

**Authors:** Sanjukta Sahoo, Tara P Tripathy, KC Pradheep Kumar, Siva Priya, Arthi Ganapathy, Praveen K Ravi

**Affiliations:** 1 Anatomy, All India Institute of Medical Sciences, Bhubaneswar, IND; 2 Radiology, All India Institute of Medical Sciences, Bhubaneswar, IND; 3 Anatomy, All India Institute of Medical Sciences, New Delhi, IND

**Keywords:** lateral wall thickness, maxillary sinus, morphometry, residual bone height, sinus augmentation surgery

## Abstract

Introduction

Sinus augmentation surgery, also called sinus graft or lift, involves elevating the Schneiderian membrane in the posterior maxilla to enhance alveolar bone height by placing a bone graft or osteogenic materials. This procedure is commonly performed to support dental implants. This study evaluates the morphometry of the maxillary sinus across different age groups within the Eastern Indian population and its implications for sinus augmentation surgery.

Methods

We analysed 85 patients (55 males (64.7%) and 30 females (35.3%)) who underwent computed tomography scans, assessing key parameters such as residual bone height, lateral wall thickness, medial wall thickness, and the sinus angle. Data were summarised and presented as mean ± SD for continuous variables and frequencies for categorical data. Inferential tests were used to analyse the parameters, with a significance level of p<0.05.

Results

Our findings indicate that 56.47% (n=48) of patients had a residual bone height of ≤5 mm, necessitating sinus augmentation surgery before dental implantation. While lateral wall thickness was an important factor in selecting the appropriate surgical approach, with 16.67% (n=8) requiring the lateral window technique and 83.34% (n=40) better suited for the crestal approach, no significant gender differences were observed. However, the need for sinus augmentation surgery was more common in the 21-40 age group (p=0.008). Notably, left-side residual bone height was significantly lower than right-side residual bone height, indicating lateral asymmetry.

Conclusion

This study emphasizes the importance of maxillary sinus morphometry in optimizing sinus augmentation surgery techniques tailored to individual anatomical variations to improve surgical outcomes in the Eastern Indian population.

## Introduction

The maxillary sinus (MS) is the first developed, largest paranasal sinus located within the body of the maxilla [1]. The MS is the most clinically significant paranasal sinus as it drains against the gravity into the hiatus semilunaris of the middle meatus [2]. The dimension of the MS varies with age, gender, dentation status, and across races [3,4]. The volume of MS increases up to the second decade in females and the third decade in males, followed by a decrease in size in the next few decades [5]. At an old age, the volume of MS again increases during the process of edentation [4,6].

MS is one of the structures that shows a wide range of variations under physiological and pathological conditions, even across the sides of the same individual [7]. Being the prominent structure with variations, the knowledge about the variation of the MS is important for several maxillofacial, rhinal, and dentistry surgeries. One of those surgeries is the maxillary sinus elevation and augmentation, performed to improve the outcome of the dental screw fixations. Sinus augmentation surgery (SAS), otherwise known as sinus graft or lift, is a surgical procedure performed to improve the alveolar bone height of the posterior maxilla by lifting the lower sinus mucous membrane (Schneiderian membrane) and placing a bone graft or osteogenic materials. Various types of SAS procedures were performed, including crestal, lateral, or palatal window sinus lift surgery. Each method has a wide range of applicability and limitations, which will be selected based on the sinus morphometry.

Several studies have been reported in the literature, with the morphometry of the MS across various ethnic groups [3,4,8,9]. However, their implications in the selection of appropriate SAS techniques and outcomes were limited, especially in the Indian population. The present study aimed to assess age-related variations in maxillary sinus morphometry in the Eastern Indian population and evaluate their impact on the selection of sinus augmentation techniques.

## Materials and methods

A retrospective cross-sectional study was conducted jointly in the departments of anatomy and radiodiagnosis after obtaining approval from the Institutional Ethics Committee (T/IM-NF/Anatomy/23/180). The study included 85 patients who underwent computed tomography (CT) of the head and paranasal sinuses between January 2020 and December 2023 for indications unrelated to the maxillary sinus, such as headache, trauma, or other neurological complaints.

Selection criteria

Inclusion criteria were the availability of high-resolution CT scans of the paranasal region with clear visualization of maxillary sinuses in patients aged 0 to 100 years. Exclusion criteria included patients with known or radiologically evident maxillary sinus disease, prior facial surgery, trauma affecting sinus anatomy, congenital craniofacial anomalies, or poor image quality.

Sampling method

A purposive sampling technique was used, wherein suitable cases were identified from the hospital's picture archiving and communication system (PACS) based on inclusion and exclusion criteria.

**Figure 1:**
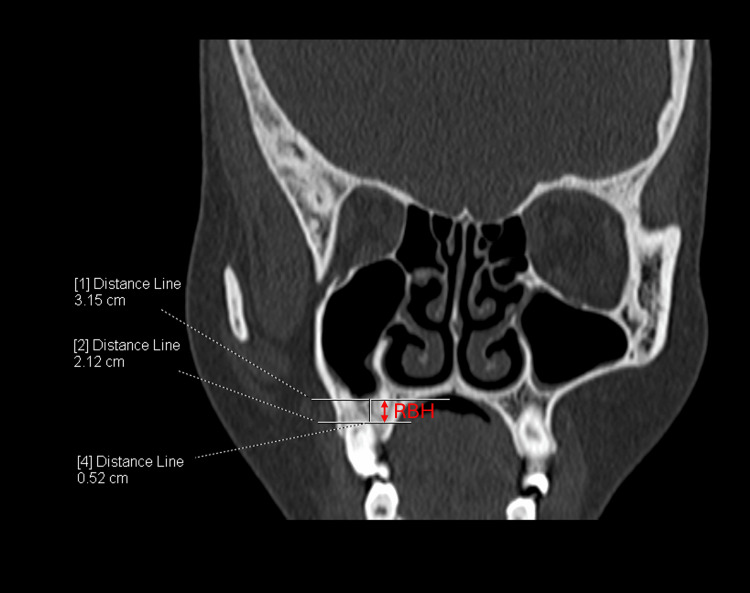
CT section showing the measurement of residual bone height (RBH) in the right maxillary sinus. The RBH is measured as the vertical distance from the alveolar ridge crest to the lowest point of the sinus floor in the coronal section.

**Figure 2:**
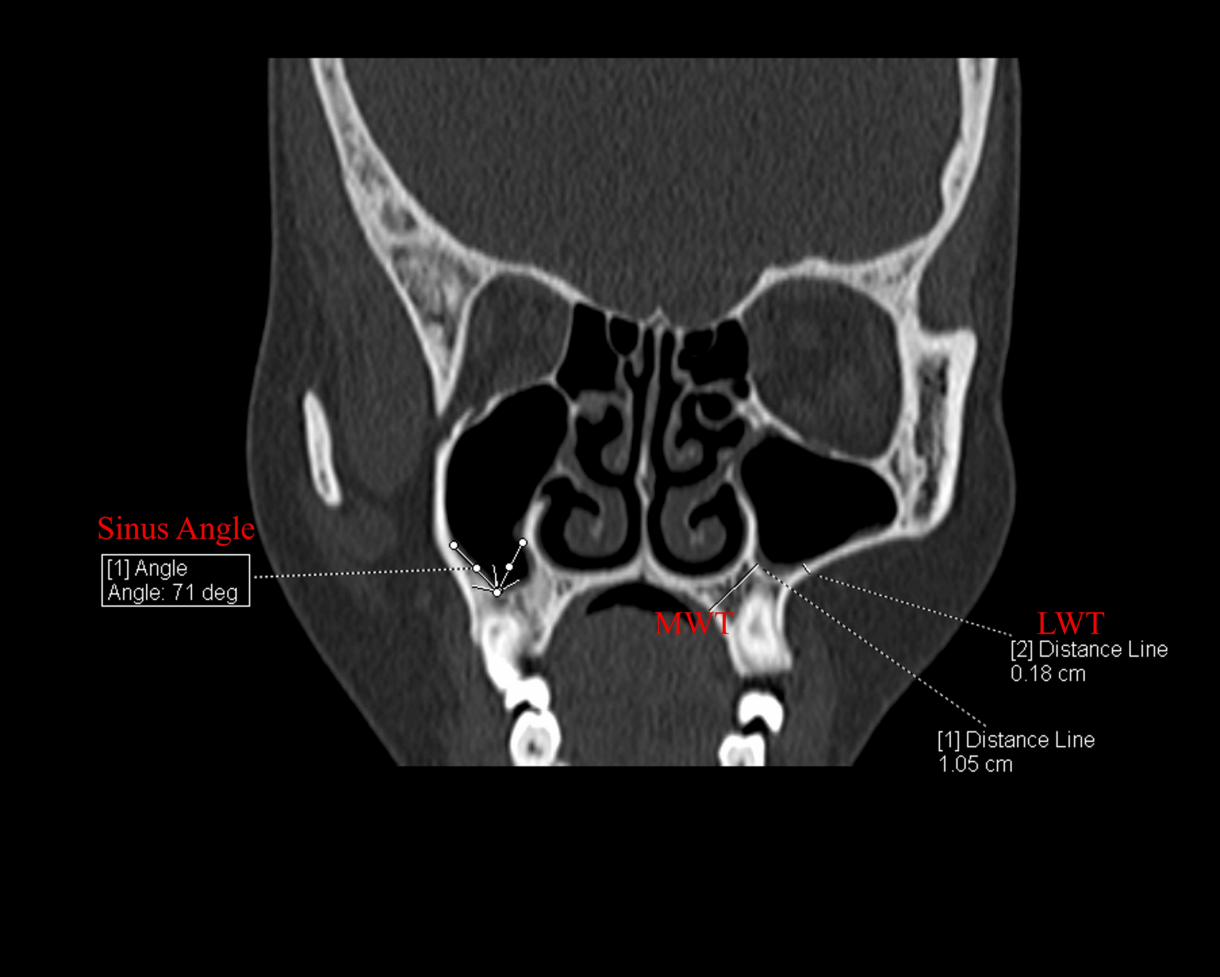
CT section showing the measurement of medial wall thickness (MWT) and lateral wall thickness (LWT) in the left maxillary sinus. Additionally, the sinus angle in the right maxillary sinus is measured in the coronal section, showing the angle formed by the intersection of the medial and lateral walls.

All CT scans were performed using a 256-slice multi-detector computed tomography (MDCT) scanner (SIEMENS SOMATOM Definition Flash, Germany). Post-processing and measurements were performed using Syngo.Via software (Siemens Healthcare, Germany). Image analysis was carried out independently by two anatomists under the supervision of a senior radiologist. For all measurements, the average of the two observers' values was used for analysis. The anatomical parameters assessed are detailed in Table 1 and illustrated in Figure 1 and Figure 2.

**Table 1:** Parameters and landmarks used to measure the morphometry. All the parameters are measured in coronal view of posterior maxillary sinus.

Parameter	Landmark	Method used	Significance
Sub-sinus residual bone height (RBH) (mm)	The distance from the alveolar ridge crest to the floor of the maxillary sinus	Measurements were taken from the highest point of the alveolar ridge to the lowest point of the sinus floor at the designated implant site.	This measurement assessed the vertical bone available for dental implant placement
Thickness of the lateral walls (LWT) (mm)	The lateral wall of the maxillary sinus.	The thickness of the lateral wall was measured from the outer cortical bone to the sinus membrane.	This measurement indicated the support available from the lateral sinus wall for augmentation and implant stability
Thickness of the palatal walls (MWT) (mm)	The medial or palatal wall of the maxillary sinus	The thickness of the palatal wall was measured from the outer cortical bone to the sinus membrane.	This assessment determined the bone volume available for grafting and implant placement
Angle of mediolateral walls (Sinus angle) (°)	The angle between the medial and lateral walls of the sinus	CT scans were used to measure the angle formed by the intersection of lines along the medial and lateral walls of the sinus.	This measurement helped in understanding the sinus anatomy and planning the surgical approach by evaluating wall inclinations

The data were summarized using mean ± standard deviation (SD) for continuous variables and frequencies for categorical variables. Inferential statistical tests included paired t-tests for right-left comparisons, independent t-tests for gender differences, and chi-square tests for associations between categorical variables (e.g., age group and SAS requirement). A p-value of <0.05 was considered statistically significant. Statistical analyses were performed using SPSS Statistics for Windows, Version 25 (Released 2017; IBM Inc., Armonk, New York).

## Results

The morphometry data of 85 patients, comprising 55 males (64.7%) and 30 females (35.3%), were included in this study. The mean age of the individual is 38.4 ± 18.3 years. The summary of the case distribution was tabulated in Table 2. 

**Table 2:** Distribution of cases requiring sinus augmentation surgery prior to dental implantation by age and gender

Age Group (years)	Male (n=55)	Female (n=30)	Total cases (n=85)	Bilateral sinus augmentation surgery required based on residual bone height (n)	Unilateral sinus augmentation surgery required based on residual bone height (n)
0-20	8	5	13	4	4
21-40	25	6	31	4	17
41-60	19	11	30	4	7
61-80	7	3	10	2	6
81-100	0	1	1	0	0

Residual bone height (RBH)

RBH is the crucial factor that determines the need for the SAS prior to the dental implants. The mean RBH on the right and left side was 7.94 ± 5.37 mm and 6.65 ± 4.36 mm, respectively. A paired t-test showed a significant difference between right RBH and left RBH (p=0.02), indicating lateral asymmetry in RBH. The majority of the cases (56.47%) (n=48) have the RBH of 5 mm or less on either or both sides, indicating the potential need for SAS. Out of which 14 cases (29.17%) had less than 5 mm of RBH bilaterally and 34 cases (70.83%) had unilaterally. 

Lateral wall thickness (LWT)

The thickness of the lateral wall of the maxillary sinus is crucial in selecting the appropriate surgical technique for SAS. The mean LWT of the maxillary sinus on the right and left side was 2.48 ± 1.04 mm and 2.76 ± 2.38 mm, respectively. There was no statistically significant difference in the laterality (p=0.312, paired t-test). In patients requiring SAS, eight cases (16.67%) had an LW of 1.5 mm or less, making them suitable for the lateral window technique, while the remaining 40 cases (83.33%) had an LW greater than 1.5 mm, suggesting the crestal approach might be more appropriate. Out of eight cases, one case (2.08%) was suitable for the bilateral lateral window approach. 

Mediolateral wall thickness (MWT)

Mediolateral wall thickness (MW) is important for understanding the structural integrity of the sinus cavity. The mean MWT of the maxillary sinus on the right and left side was 6.85 ± 3.92 mm and 6.45 ± 3.65 mm, respectively. A paired t-test revealed no significant difference between the two sides (p=0.226). Although MW is not directly used in deciding the surgical technique for SAS, it provides additional context regarding anatomical variations of the maxillary sinus.

Sinus angle

The angle between the lateral and medial walls of the maxillary sinus (sinus angle) is a critical parameter influencing surgical access during SAS. The mean sinus angle of the right and left sides was 91.18° ± 15.02° and 89.70° ± 18.33°, respectively. The difference between these angles was not statistically significant (p=0.477, paired t-test). While the sinus angle is not a primary criterion for determining SAS, extreme angles could complicate the surgical approach.

Comparison of morphometric parameters by gender

An independent t-test was conducted to compare morphometric parameters between males and females. The results are summarized in Table 3. No significant differences were observed between males and females for RBH, LWT, MWT, or right sinus angle, suggesting that these parameters are relatively consistent across genders. However, only the left sinus angle shows significantly more in males when compared to females, with p=0.009.

**Table 3:** Gender comparison of morphometric parameter RBH - residual bone height; LWT - lateral wall thickness; MWT - medial wall thickness * p<0.05

Parameter	Males (n=55)	Females (n=30)	p-value
Right RBH (mm)	8.05 ± 5.33	7.70 ± 5.57	0.790
Left RBH (mm)	6.87 ± 4.28	6.14 ± 4.58	0.493
Right LWT (mm)	2.45 ± 0.99	2.53 ± 1.18	0.772
Left LWT (mm)	2.89 ± 2.71	2.47 ± 1.33	0.338
Right MWT (mm)	7.04 ± 4.15	6.42 ± 3.38	0.471
Left MWT (mm)	6.28 ± 3.57	6.85 ± 3.85	0.518
Right sinus angle (°)	90.90 ± 14.83	91.81 ± 15.70	0.804
Left sinus angle (°)	93.10 ± 15.47	81.99 ± 22.02	0.009*

Need for sinus augmentation surgery (SAS)

Based on the morphometric analysis, 48 cases (56.47%) were identified as requiring SAS due to insufficient RBH (≤5 mm). Among these, the distribution of the required surgical techniques was as follows: eight cases (16.67%) of lateral window technique were indicated for this approach due to an LW of 1.5 mm or less. Out of which one is bilateral (2.08%). Crestal approach: 40 cases (83.34%) were better suited for this technique, with an LW greater than 1.5 mm. The requirement for sinus augmentation surgery (SAS) was significantly more prevalent in the 21-40-year age group (χ²(3)=10.84, p=0.013, Cramér's V=0.34). Post-hoc comparisons revealed this difference was specifically significant between the 21-40 group (67.7% prevalence) and the 41-60 group (36.7% prevalence; χ²(1)=6.93, p=0.008, φ=0.33) with no significant gender-based differences. This reflects the increased likelihood of early or moderate alveolar bone resorption in these populations.

## Discussion

In elderly patients, tooth loss often leads to the resorption of the alveolar ridge and the pneumatization of the maxillary sinus. These changes can significantly reduce the likelihood of successful outcomes in dental rehabilitation surgeries. To enhance the effectiveness of these rehabilitation procedures, SAS is frequently performed, particularly when the RBH is less than 5 mm. The present study aims to evaluate the morphometry of the maxillary sinus across various age groups within the Indian population, thereby providing valuable insights into the necessity and optimal selection of SAS tailored to this demographic.

Research indicates that SAS can achieve a remarkable success rate, reportedly reaching 100%, with graft survival extending up to three years during follow-up. This success is contingent upon the careful selection and execution of the surgical technique, which should be informed by the anatomical morphometry of the maxillary sinus [10]. Several anatomical parameters are critically evaluated prior to surgical planning, including RBH, LWT, MWT, sinus angle, sinus membrane thickness, and the presence, direction, and height of sinus septa. Additionally, factors such as the type of edentulism, the palatonasal recess angle, and the diameter of the alveolar antral artery also play essential roles in preoperative assessments [9,11-13]. Notably, Lyu et al. emphasized the importance of considering sinus health alongside anatomical factors in the decision-making process for SAS [11].

In our study, we observed that the RBH is significantly lower on the left side compared to the right, a finding that aligns with previous research conducted on the Korean population [14]. However, our results also indicate that there is no significant variation in RBH between the right and left sides, which corroborates existing literature. It is important to note that most studies focusing on the morphometry of the maxillary sinus in the Indian population have primarily documented parameters such as volume, height, surface area, and width [8,15-18]. To the best of our knowledge, our study is pioneering in documenting the RBH and elucidating its clinical significance in the context of SAS. 

While this study provides valuable insights into maxillary sinus morphometry and its implications for SAS, certain limitations should be acknowledged. The sample size, though adequate for preliminary analysis, was confined to a single geographic region (Eastern India), which may not fully represent the broader Indian population. The retrospective design, while efficient, relies on pre-existing imaging data, which may introduce variability in scan quality and patient selection. Although inter-observer agreement was ensured by averaging measurements between two anatomists, subtle discrepancies in landmark identification could persist. The cross-sectional nature of the study also limits the ability to assess age-related changes over time. Finally, while morphometric parameters were correlated with SAS suitability, clinical outcomes such as postoperative complications or graft success rates were not evaluated.

## Conclusions

This study provides a comprehensive evaluation of the morphometric variations of the MS across different age groups within the Eastern Indian population. Our findings reveal significant anatomical variations in the MS, which play a crucial role in determining the appropriate technique for SAS. These variations, particularly in parameters such as RBH, LWT, and MWT, must be carefully considered during preoperative planning to ensure optimal surgical outcomes. The results also highlight the importance of tailoring SAS procedures, such as crestal or lateral window techniques, based on individual sinus anatomy. This personalized approach can enhance the success rate of SAS by addressing the specific anatomical challenges presented by the patient's sinus morphology.
